# Mechanistic role of quercetin as inhibitor for adenosine deaminase enzyme in rheumatoid arthritis: systematic review

**DOI:** 10.1186/s11658-024-00531-7

**Published:** 2024-01-16

**Authors:** Amira Atta, Maha M. Salem, Karim Samy El-Said, Tarek M. Mohamed

**Affiliations:** Biochemistry Division, Chemistry Department, Faculty of Science, Tanta University, Tanta, 31527 Egypt

**Keywords:** Rheumatoid arthritis, Adenosine deaminase, Flavonoid, Quercetin, Synovial fluid

## Abstract

Rheumatoid arthritis (RA) is an autoimmune disease involving T and B lymphocytes. Autoantibodies contribute to joint deterioration and worsening symptoms. Adenosine deaminase (ADA), an enzyme in purine metabolism, influences adenosine levels and joint inflammation. Inhibiting ADA could impact RA progression. Intracellular ATP breakdown generates adenosine, which increases in hypoxic and inflammatory conditions. Lymphocytes with ADA play a role in RA. Inhibiting lymphocytic ADA activity has an immune-regulatory effect. Synovial fluid levels of ADA are closely associated with the disease’s systemic activity, making it a useful parameter for evaluating joint inflammation. Flavonoids, such as quercetin (QUE), are natural substances that can inhibit ADA activity. QUE demonstrates immune-regulatory effects and restores T-cell homeostasis, making it a promising candidate for RA therapy. In this review, we will explore the impact of QUE in suppressing ADA and reducing produced the inflammation in RA, including preclinical investigations and clinical trials.

**Graphical Abstract:**

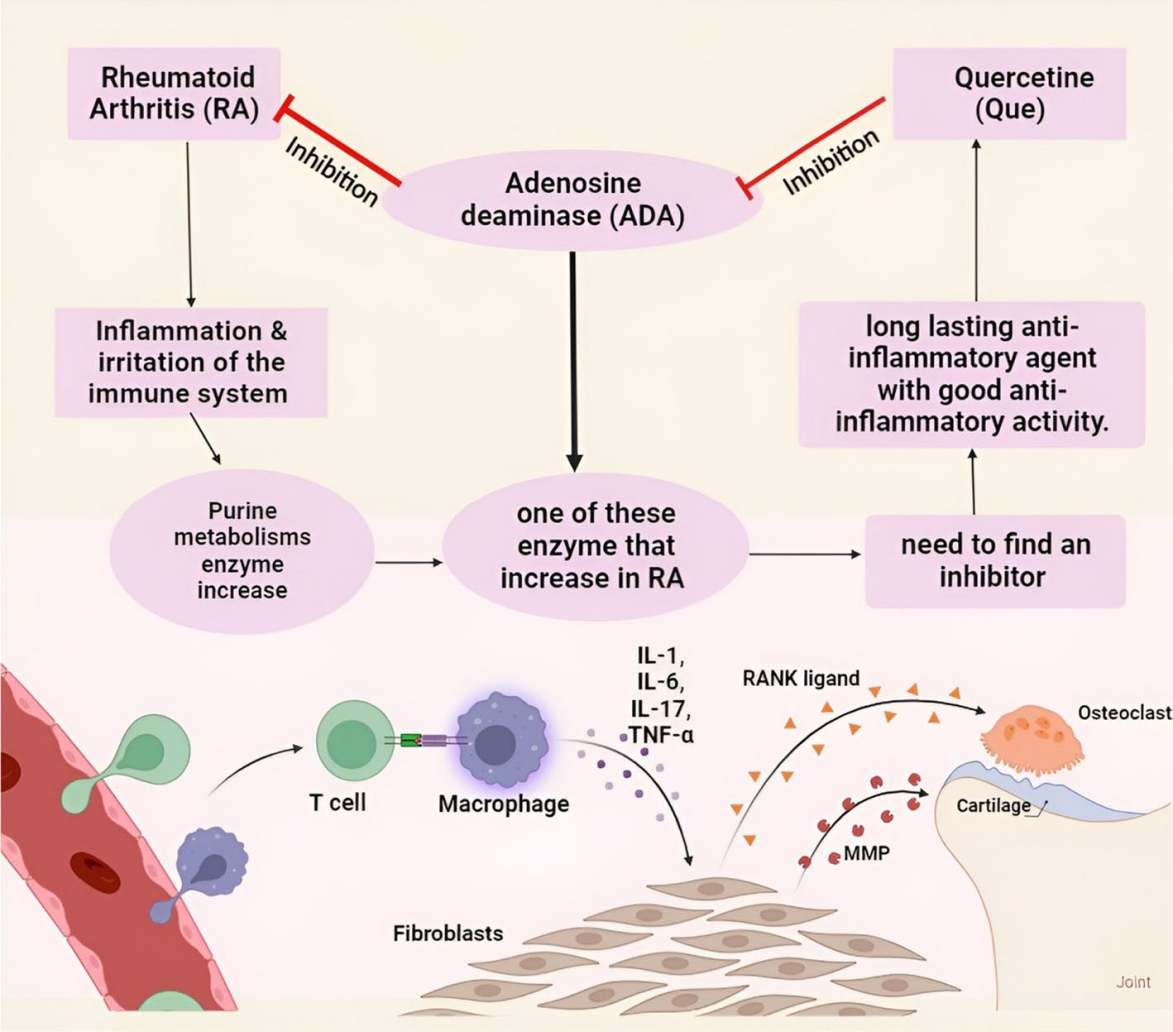

## Rheumatoid arthritis (RA)

**Fig. 1:**
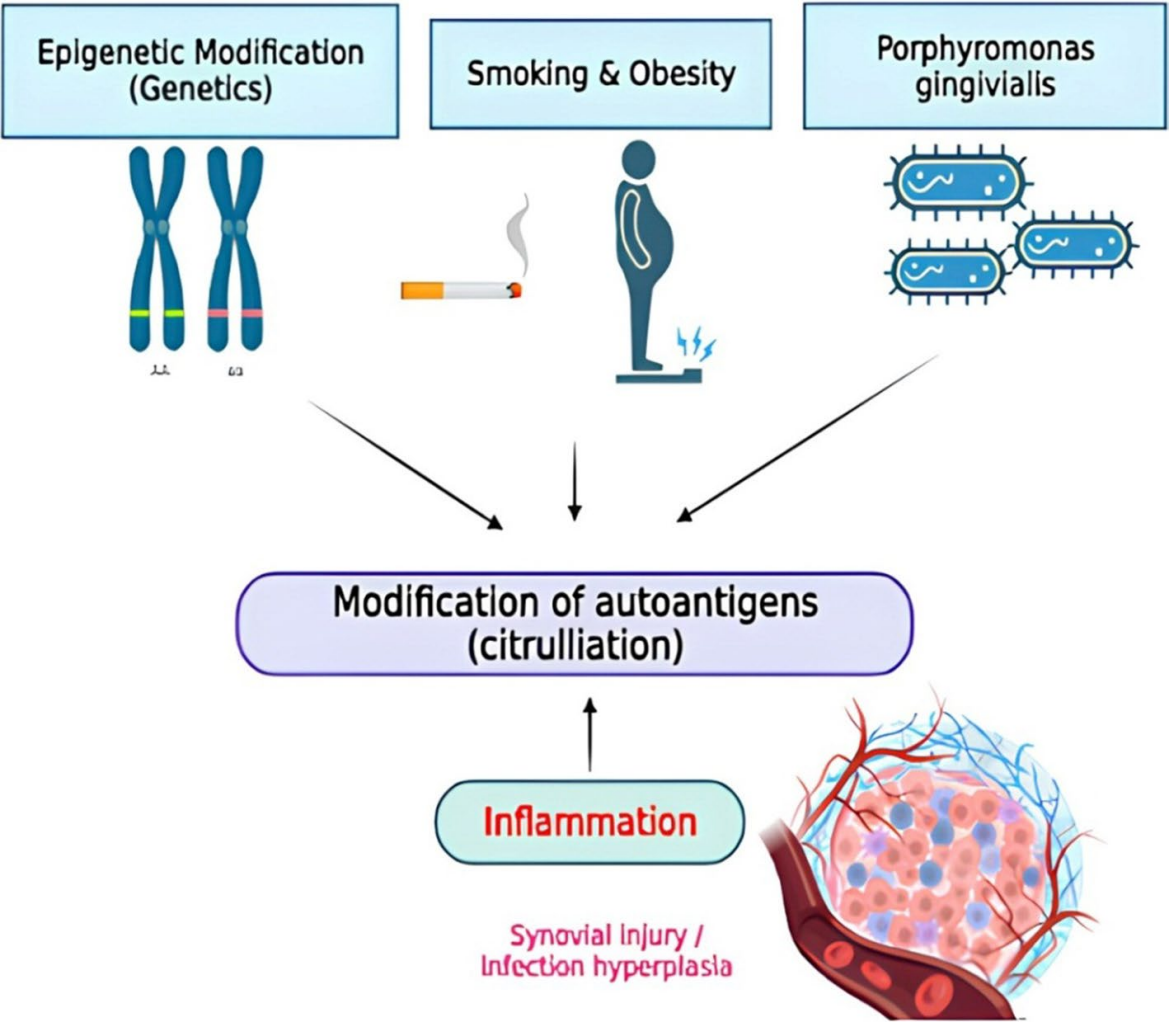
Factors that contribute to the progression of RA

Rheumatoid arthritis (RA) is a chronic inflammation of the joints. The activation of synovial tissue in the joint capsule, cartilage and bone invasion, as well as increasing joint dysfunction are the key hall marks of RA [1]. The epigenetic and environmental variables have a role in the genesis and progression of RA. Furthermore, non-genetic variables such as sex hormones, smoking, periodontal infection, and microbiota, as well as autoantibodies, cytokines, chemokines, and proteases, are engaged in the inflammatory processes that assault the cartilage and bone, resulting in joint dysfunction [2] (Fig. 1). The over-activation of T and B lymphocytes, synovial-like fibroblasts, and macrophages, as well as the significant production of proinflammatory cytokines like tumor necrosis factor alpha (TNF-α) and interleukin 6 (IL-6), are the main inflammatory processes that result in ongoing inflammation and joint degeneration, as mentioned by Huang et al. [1].

### Epidemiology

**Fig. 2:**
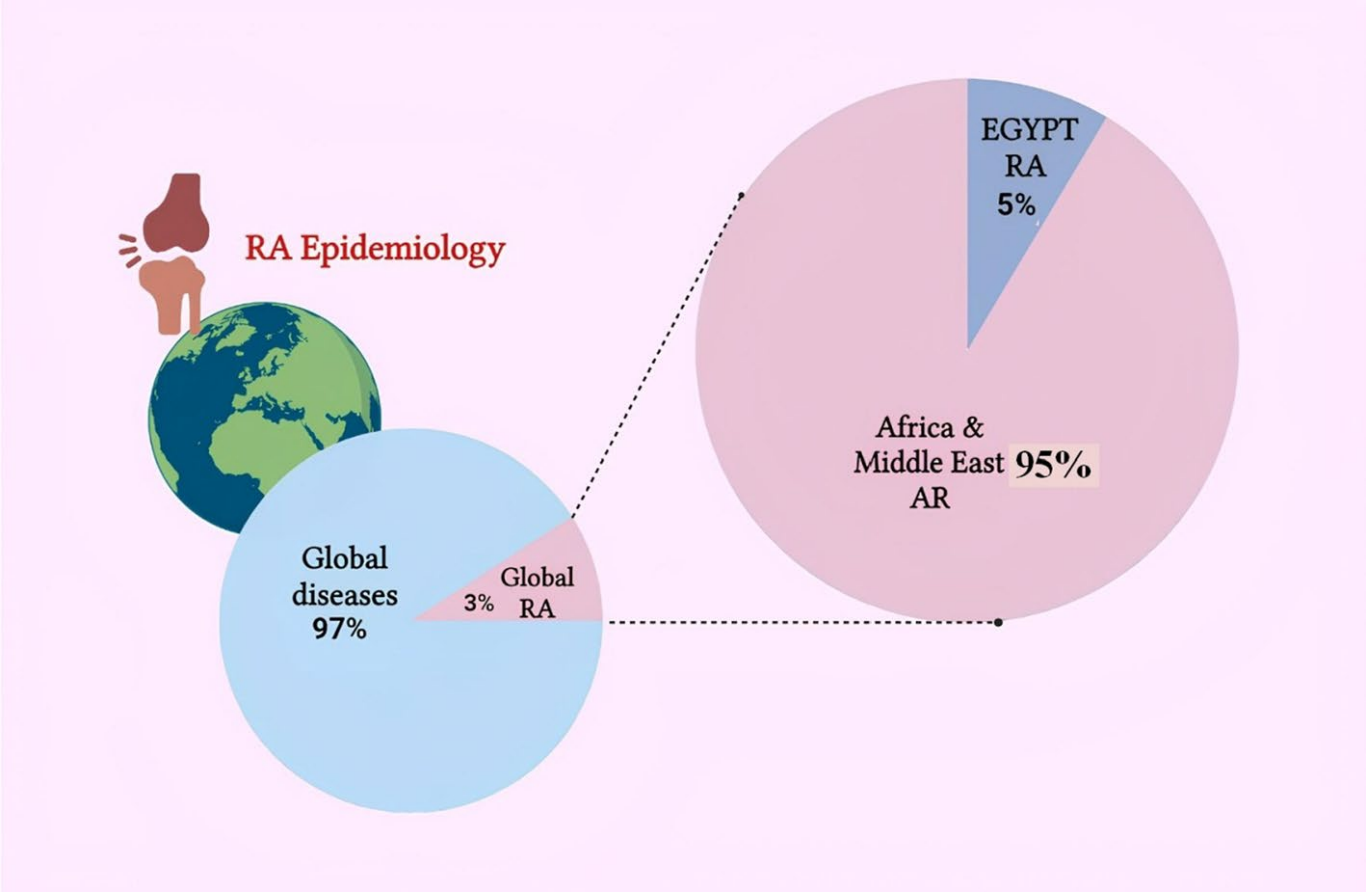
The epidemiology of rheumatoid arthritis

RA is present worldwide, its prevalence varies among countries, regions, and ethnic groups [3]. The prevalence of RA is higher in Africa and the Middle East, usually ranging from 0.25% to 3.4% in most countries, according to Finckh et al. [4]. Also, Abdel Fattah et al. [5] estimated that the RA disease prevalence in Egypt is about 5%. These estimates are based on older populations, self-reported patients, and clinic or hospital-based studies [6], and urban populations tend to be higher than those based on data from the Global Burden of Disease (GBD) study, as mentioned by Riedmann et al. [7] (Fig. 2).

### Causes of RA

**Fig. 3:**
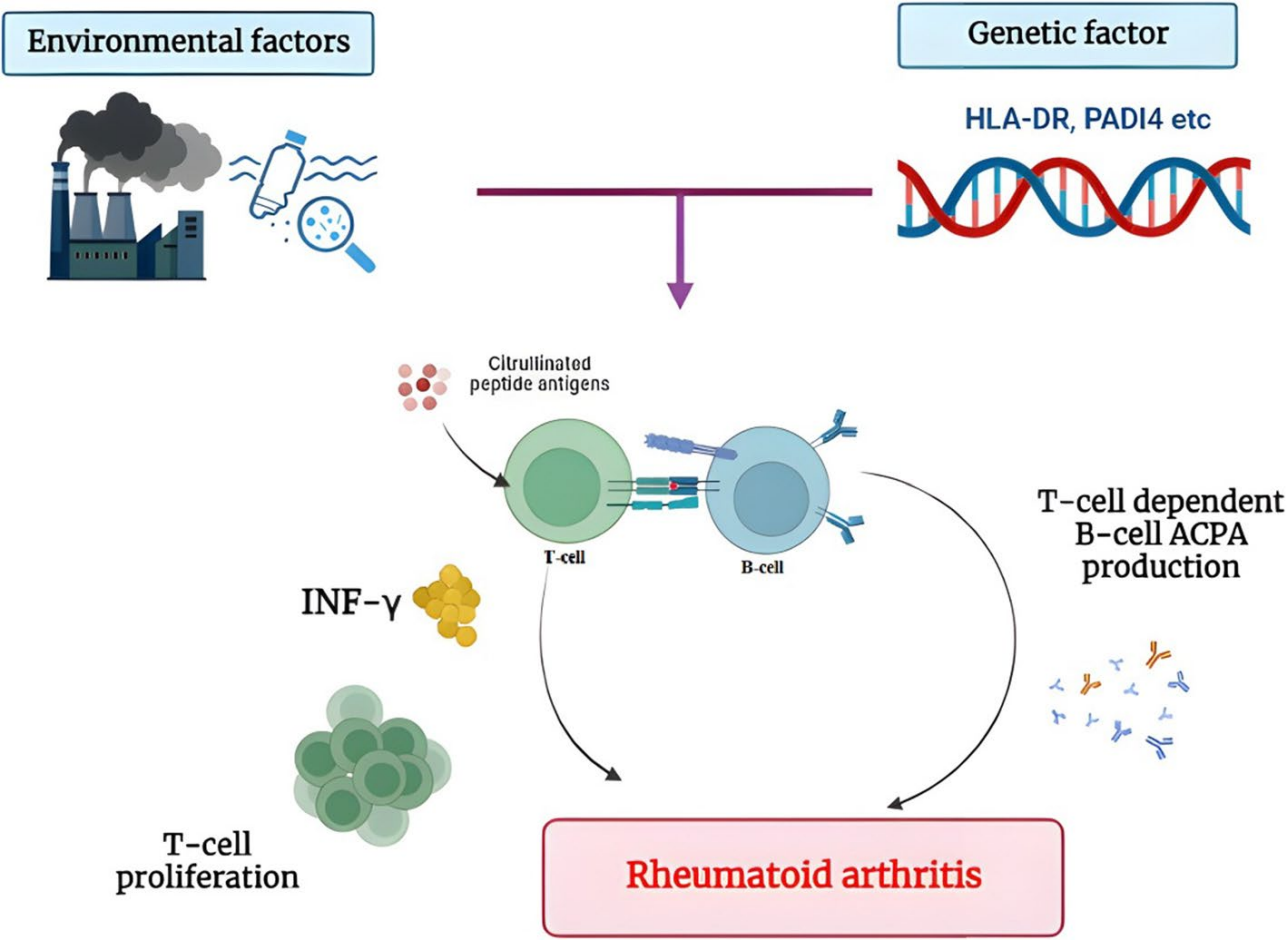
Causes of rheumatoid arthritis

While the cause of RA is unknown, there is evidence that both hereditary and environmental factors have a role in the disease’s progression [4] (Fig. 3). The patient’s genetic predisposition, which results in the production of auto reactive T and B cells, and a triggering event, such as a viral or bacterial infection or tissue injury, are the two independent factors that lead to the initial cause of RA. Furthermore, RA is most likely caused by a stochastic event caused by a combination of genetic variation, epigenetic changes, and environmental variables in people who are genetically vulnerable to the disorder [8]. Moreover, the cause of RA has been linked to lung microbiota, periodontal disease (periodontitis), and infections [9]. Identical twins had higher concordance risk rates than unrelated control groups and non-identical twins, suggesting that genetic factors have a role in the development of RA. A family history of RA raises the likelihood of developing the disease by three to five times [10].

Another cause of RA is the discovery of over 100 loci related with disease progression in genome-wide association studies using single nucleotide polymorphisms (SNPs) [11]. Many of these loci are seen in other chronic inflammatory diseases and are implicated in the regulation, activation, and maintenance of immune responses [12]. *Human leukocyte antigen *(HLA) alleles, which are linked to an increased risk of developing RA, are among these sites [13]. Furthermore, HLA variants have been associated to more severe bone deterioration and higher death rates [14].

### Path mechanism of RA

**Fig. 4:**
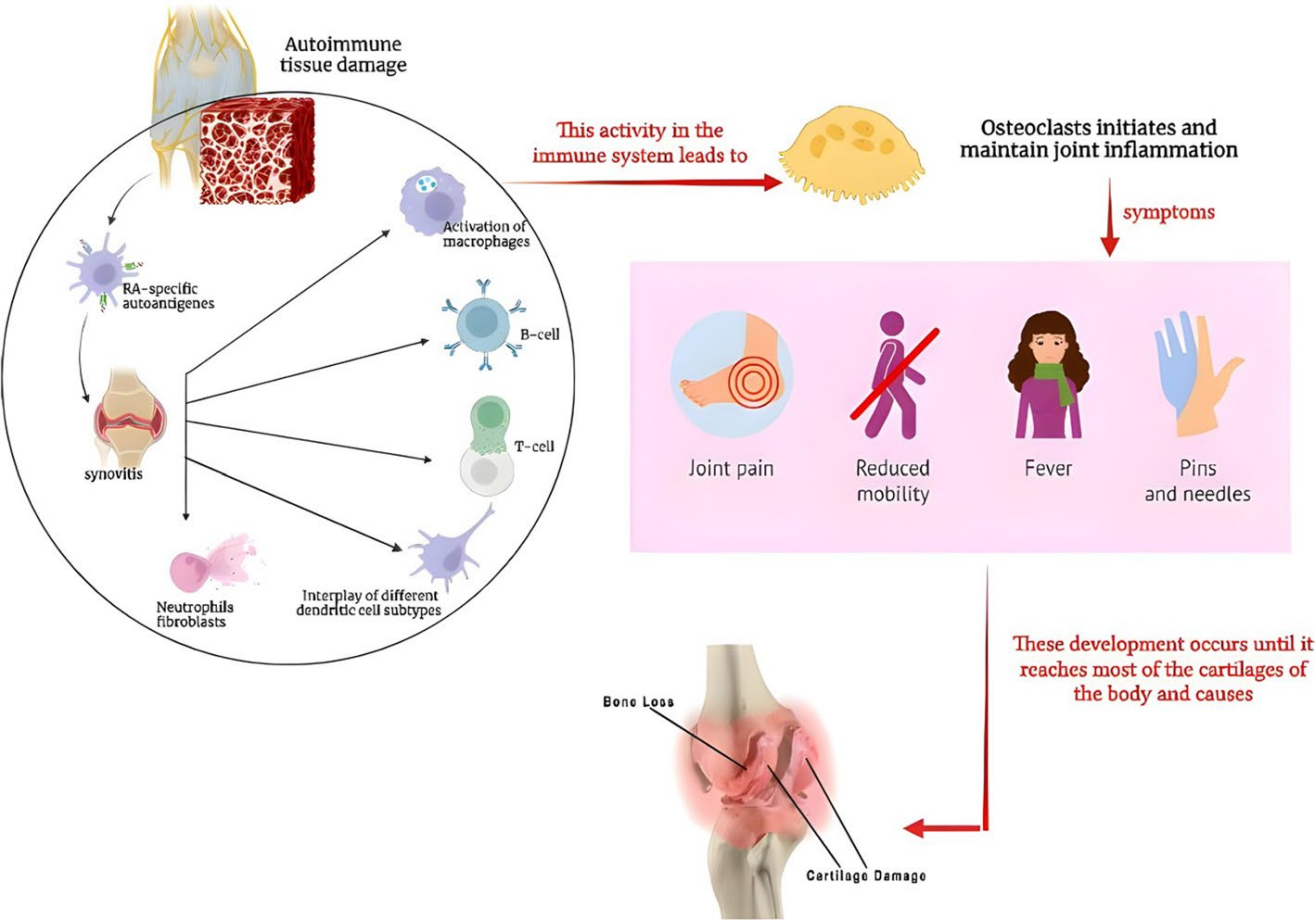
Path mechanism of rheumatoid arthritis

Synovitis, an inflammation of the joint capsule that affects the accompanying bones, the synovial membrane, and the synovial fluid, is an indicator of autoimmune tissue destruction in RA [15]. A variety of dendritic cell subtypes, T cells, macrophages, B cells, neutrophils, fibroblasts, and osteoclasts collaborate to initiate and sustain joint inflammation [16]. Due to the frequency of RA-specific autoantigens and the difficulty to totally remove them, continuing immune cell activation leads to a self-perpetuating inflammatory state in the joint, causing pain and joint swelling in afflicted individuals [17] (Fig. 4). Pannus, which is a swelling of the synovial membrane that invades the periarticular bone at the cartilage–bone interface, is caused by the arthritic joint’s continuing inflammatory milieu and results in bone loss and cartilage deterioration [18].

### Dendritic cells (DC) and RA

DCs are crucial for RA inflammation maintenance and promotion [19]. DCs, or antigen-presenting cells, play an important role in the initiation of immune responses by capturing and presenting antigens to T cells [20]. DCs of the myeloid (mDCs) and plasmacytoid (pDCs) subtypes have been found in the synovial tissue of patients with RA [21]. The high density of DCs in the synovium of patients with RA synovium shows that DCs play an important role in the etiology of RA [22]. There is evidence that T-cell responses in the synovium are improved by DCs, which may contribute to the pathophysiology of RA [23]. DCs are present in both inflammatory and homeostatic tissue. DCs are drawn from the blood into the synovium in RA [24]. The proinflammatory cytokines TNF-α, interleukin (IL)-1, and IL-6 are generated by both inflamed synovial lining cells and invading immune cells and can further encourage DCs to cause inflammation [16].

The generation of autoantibodies is aided by DCs’ involvement in B-cell activation. DCs also present antigen, release cytokines, and increase co-stimulatory molecules, which all serve to excite B cells. Autoantibodies are produced because of the increased B cell activity, which helps to cause RA [25]. Through interactions with adhesion molecules produced on endothelial cells, DCs can aid in the attraction of other immune cells, such as T cells. In RA synovial tissue, DCs have also been demonstrated to be resistant to apoptosis [17]. This ability to resist apoptosis enables DCs to stay in the synovium, helping to maintain synovitis and encouraging the release of proinflammatory cytokines. In addition, it has been demonstrated that DCs express more toll-like receptors (TLRs) in RA synovial tissue than in normal control tissue [26].

**Fig. 5:**
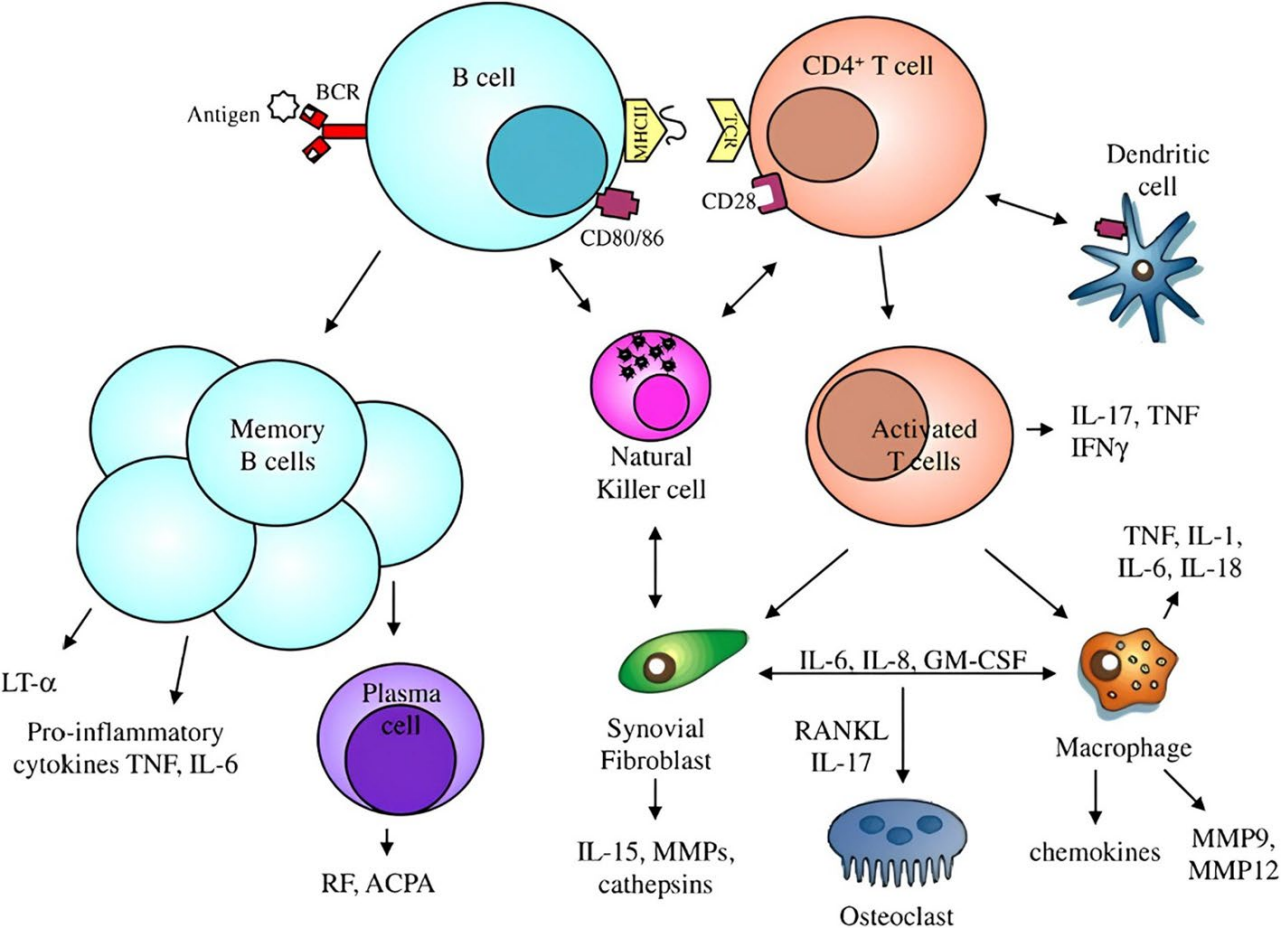
Dendritic cells the main cause for maintenance of inflammation in rheumatoid arthritis

DCs generate cytokines and chemokines and exhibit surface chemicals that regulate the immune system’s induction, activation, and maintenance of tolerance [27]. However, due to the changes in DC activity and distribution, RA and other autoimmune diseases can also result in autoimmune inflammation [28]. Changes in DCs are thought to be the primary cause of RA, increasing DC migration to the inflamed joint [29]. The upregulation of CCR6, a chemokine CCL20 receptor on DCs, is assumed to be the source of DC attraction to synovial tissue [30]. Once they have grown in the joint, DCs produce cytokines including IL-12 and IL-23 (Fig. 5), encourage antigen-specific Th17 responses and result in an imbalance of Th1, Th2, and Th17 responses [31].

## Cytokines and inflammation

In the course of developing inflammation in RA, cytokines are significant molecules [32]. They act as a link between skin cells, immune cells, and tissue cells. Joint inflammation depends on key effector cytokines generated by T cells, including TNF-α, IL-17A, interferons (IFN), and receptor activator of nuclear factor kappa-β ligand (RANK-L) [33]. Between skin cells, immune cells, and tissue cells, they serve as a bridge. TNF-α, IL-17A, interferons, and RANK-L are important effector cytokines produced by T cells that are necessary for joint inflammation [34]. Uncontrolled inflammation and bone and cartilage degeneration are brought on by TNF-α upregulation [35]. Additionally, TNF-α induces osteocytes to produce RANK-L, which encourages osteoclastogenesis and causes cells from the monocyte/macrophage lineage to differentiate into osteoclasts [36]. TNF-α may draw leukocytes to the synovium and induce inflammation by inducing the release of inflammatory cytokines like IL-1 and IL-6 [37].

**Fig. 6:**
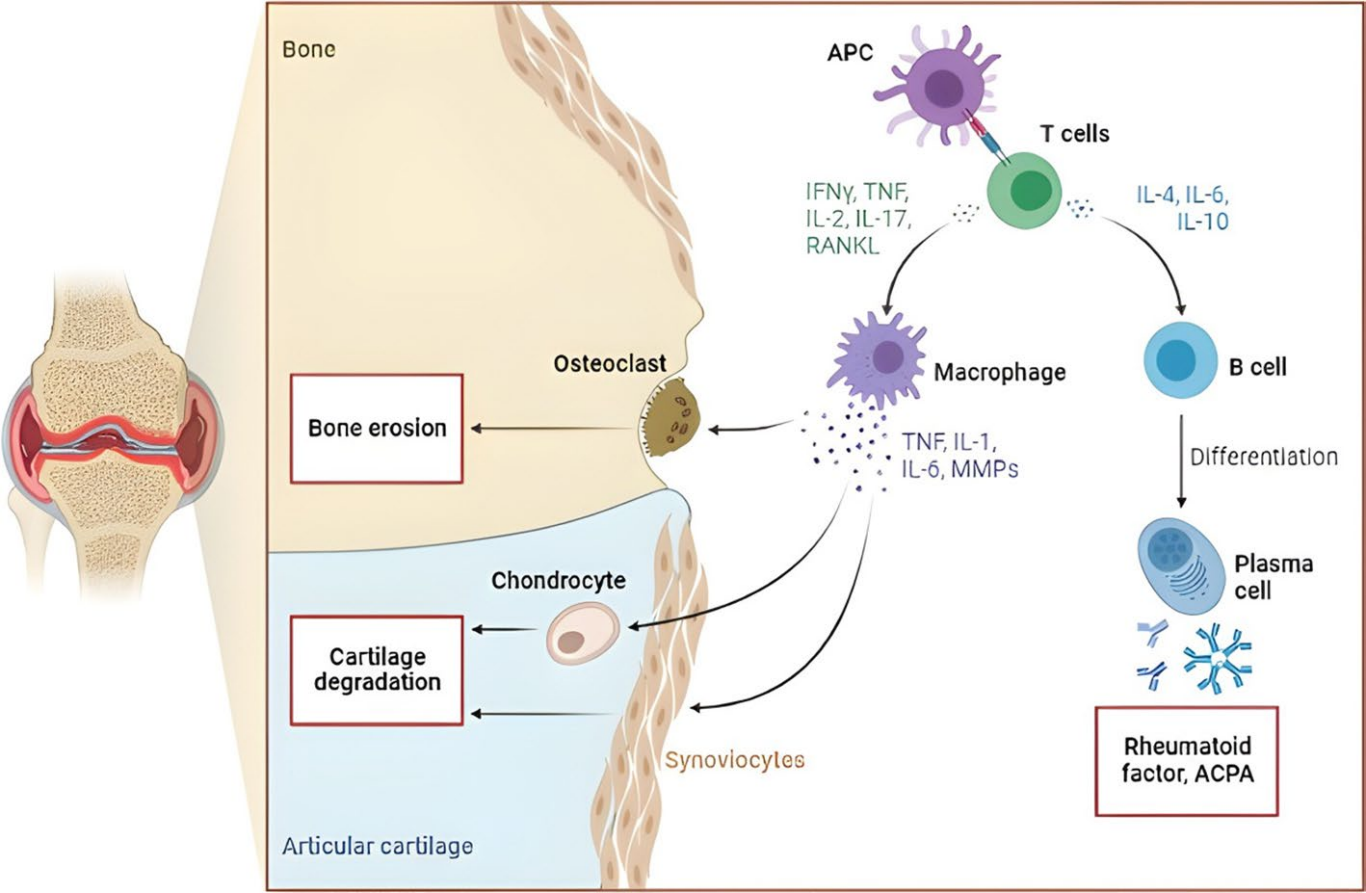
Cytokines and inflammation in rheumatoid arthritis disease

Th17 cells produce IL-17A, which causes localized inflammation and hastens the development of RA illness by accelerating the loss of cartilage, bone resorption, and angiogenesis (Fig. 6) [38]. IL-17A plays a significant role in the development of RA by stimulating the synthesis of RANK-L through osteoblasts and synoviocytes leading to decreased bone development and increased bone degradation [39]. Also, facilitating the formation of matrix metalloproteinase (MMP-1) by synoviocytes [40] and promoting both endothelial cell migration and angiogenesis [41]. Inflammation and joint degeneration brought on by activated neutrophils are exacerbated by proinflammatory cytokines produced by synovial activated macrophages that attract and excite other innate immune cells [42].

Activated fibroblasts also contribute to local joint injury by developing RANK-L and MMPs and migrating between joints [1]. Overall, the RA joint inflammation is a unique tissue reaction that combines local fibroblasts with active proinflammatory phenotypes, matrix modulation, osteoclast formation, and invasive properties [43].

## Neovascularization and RA

**Fig. 7:**
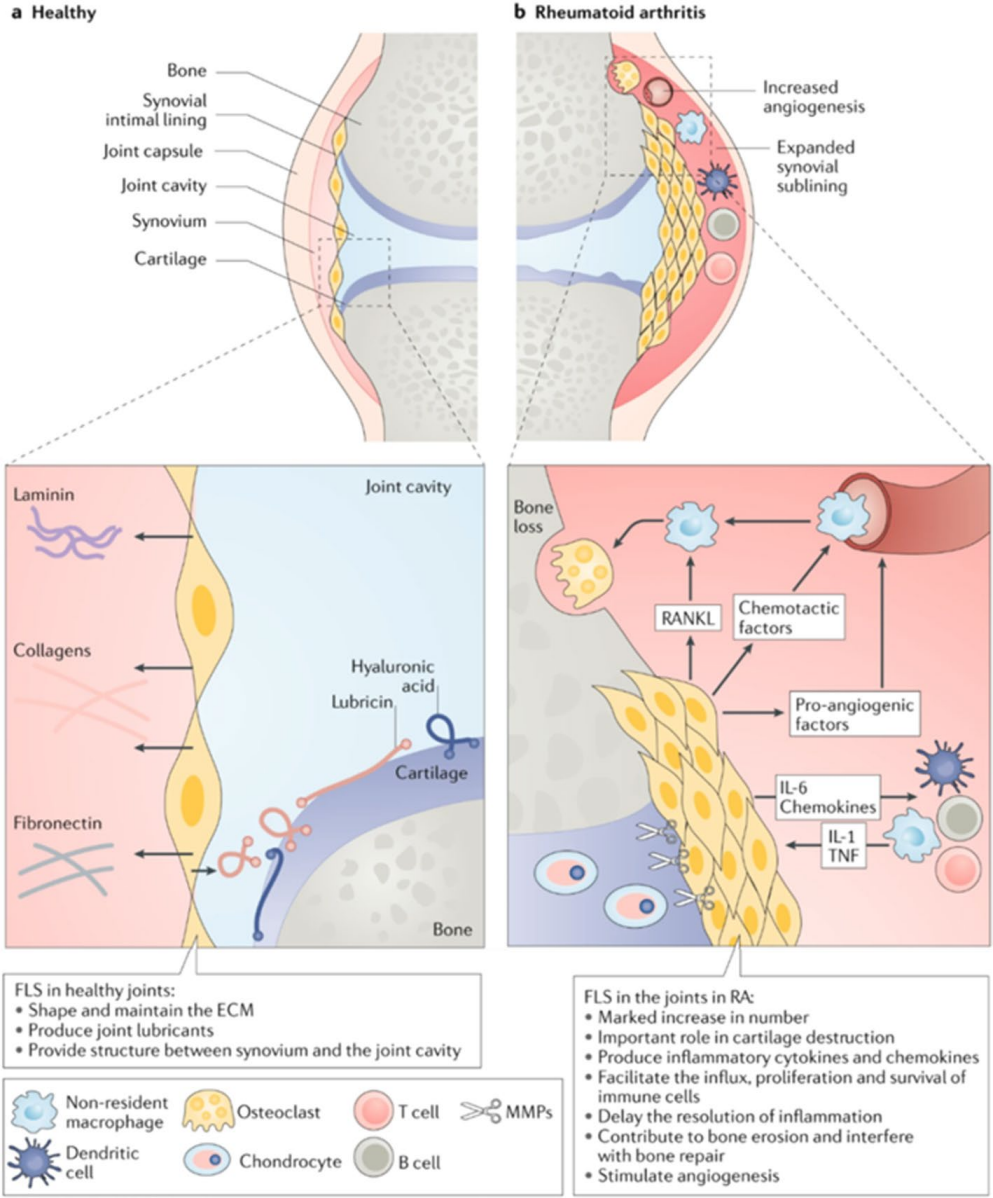
Mechanical pathway for the role of synovial neovascularization in rheumatoid arthritis

The RA prevascular stage is brief before progressing to a major vascular stage with an obvious rise in vessel formation [44]. An increase in macrophages and fibroblast synoviocytes is an indication of the prevascular stage in the lining layer [45]. The destructive and invasive front known as the synovial pannus develops when the cluster of differentiation 4 (CD4^+^) T cells, B cells, and macrophages enter the sublining layer (Fig. 7). This pannus acts like a regional cancer, invading and damaging bone and cartilage [46].

In both inflamed and non-inflamed joints, there is an increase in the synovial lining layer’s thickness and mononuclear cell infiltration [47]. The lining layer of RA synovium is chronically hypoxic despite increased vessel density associated to active endothelial growth and EC survival [48] (Fig. 7). Direct oxygen tension measurements are a spike in synovial hypoxic metabolites that supported the occurrence of reduced oxygen levels in the RA synovium [15]. The RA synovium’s vasculature is further put in danger by the mobility and accumulation of synovial fluid, which exacerbates hypoxia in a preischemic environment. The concurrent spike in metabolic demand and hypoxia serves as a strong signal for the emergence of new vascular tissue [49].

### Angiogenic

**Fig. 8:**
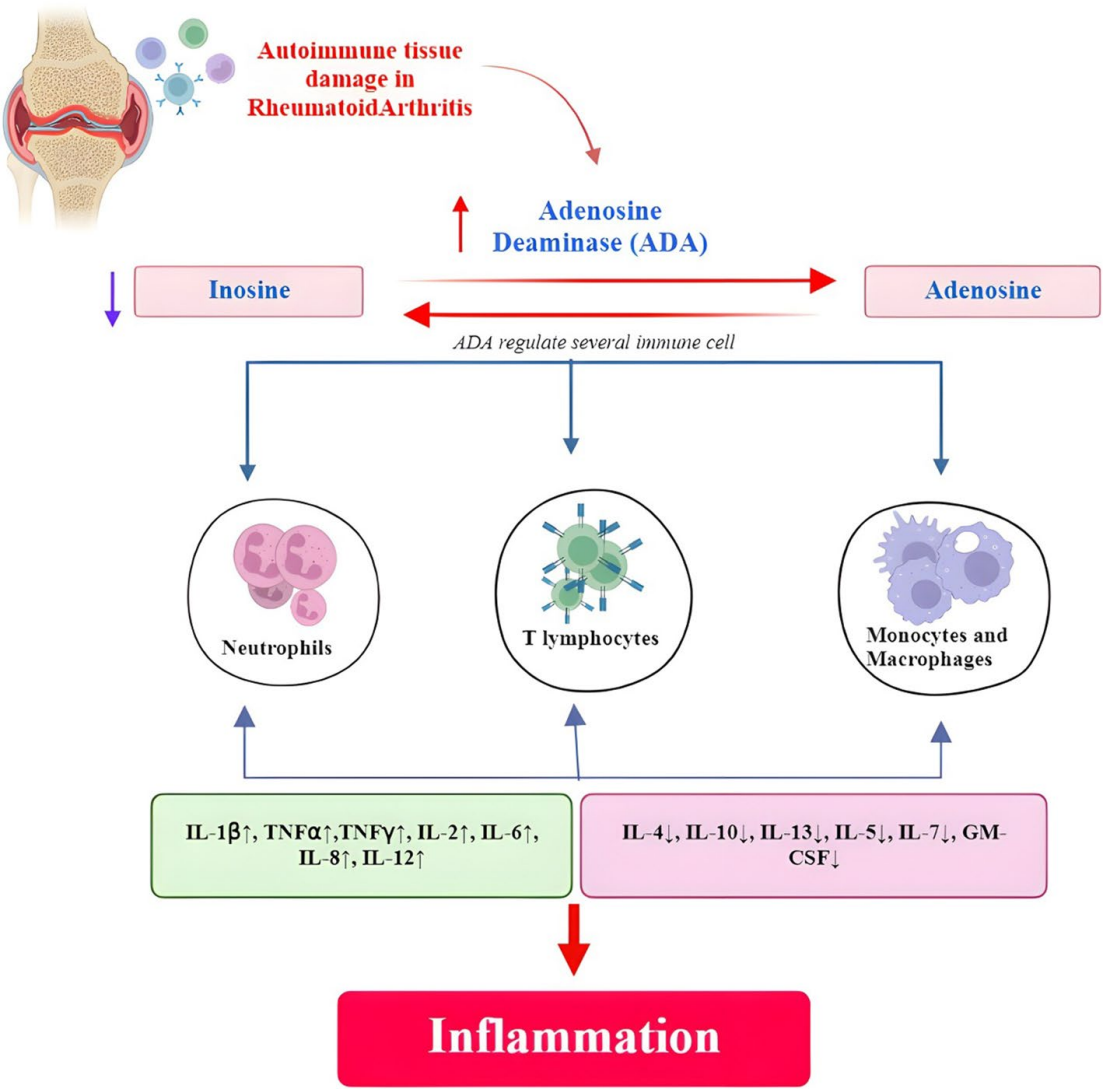
Mode of action of ADA in rheumatoid arthritis

The proinflammatory and hypoxic microenvironment leads to the production of a wide array of growth factors, cytokines, and chemokines in the RA synovium. These components cause endothelial cells (ECs) to arise from preexisting arteries, to proliferate, and to move into inflamed regions, which starts the RA vascular stage [50] (Fig. 8).

Increased angiogenesis is also linked to morphological defects in newly formed capillaries in rheumatoid arthritis. Mural cells that are positive for smooth muscle actin (α-SMA) are absent from this subgroup of immature, dilated, and leaky neoangiogenic arteries. It is believed that chronic vascular endothelial growth factor (VEGF) overexpression is due to the imbalance between EC proliferation and the absence of concomitant pericyte production. Most often found in the sublining layer, these small capillaries are surrounded by inflammatory infiltrates (Fig. 7). Unexpectedly, RA activity and progression are connected to the density of immature vasculature, which is the only vascular component to regress in response to anti-TNF therapy [51].

### Vasculogenesis

Is the process through which blood vessels grow organically. This process begins in the mammalian embryonic yolk sac and continues later during the embryo’s development [52]. In the synovial membrane area, these cells were previously shown to be present in cell clusters next to CD133^+^ cells. Alpha-chemokine receptor specific for stromal-derived factor 1 (CXCR4) was expressed in large amounts by CD34^+^ progenitor cells, whereas VEGF receptor 2 (VEGFR-2) was expressed by CD34^+^ and CD133^+^ cells. Moreover [53], CD34^+^ cells were cultured in the presence of granulocyte-macrophage colony-stimulating factor (GM–CSF) and stem cell factor after being extracted from the bone marrow of 13 patients with active RA and 9 controls. Von Willebrand factor-positive cells (vWF^+^) and CD31^+^/vWF^+^ cells were produced by RA bone marrow-derived CD34^+^ cells in much higher amounts compared to control samples. Consequently, bone marrow CD34^+^ cells may aid in the neovascularization of the synovium and may be responsible for the etiology of RA by supplying endothelial precursor cells [54].

## Clinical biomarkers and diagnosis of RA

### Clinically

There are several typical RA symptoms, including stiff and tender joints, morning joint pain, widespread nausea, and inconsistent lab results [55]. Early identification is essential in the treatment of RA since it may often stop the disease’s course in patients, preventing damage to the joints, irreversible disease progression, and early injury. RA biomarkers can be assessed at the molecular, biochemical, or cellular level and is a quantifiable sign of a particular biochemical, physiological, or morphological state. The past 5 years have seen the identification of novel biomarkers, particularly genomics, which are the fields that follow from the study of proteins (proteomics) and metabolites (metabolomics). When treating patients with RA, these indicators help the doctor choose the best course of action [56].

### Typically

To diagnose RA, a variety of methods are utilized, including the assessment of risk factors, family history, joint ultrasound sonography, assessment of laboratory indicators including high C-reactive protein (CRP) and erythrocyte sedimentation rate (ESR) in blood, discovery of RA-specific autoantibodies, and others [57]. CRP and ESR are frequently used as clinical indicators to assess the overall inflammatory status of patients with RA [58]. The acute phase reactant, often known as CRP, is composed of five 23 kDa pentraxin protein subunits. If tissue injury, inflammation, or infection are present, the serum concentration will rise by three or more log steps [59]. Increased neutrophil influx and phagocytosis are caused by CRP, which also activates the classical complement system and serves as an immune effector [60]. The release of monocyte chemoattractant protein 1 (MCP-1) and macrophage colony-stimulating factor (M–CSF), as well as the development of proinflammatory cytokines and the subsequent amplification of inflammation, have all been shown to be additional ways that CRP promotes macrophage survival and proliferation [61].

Clinical indications such morning stiffness, pain, nausea, grip strength, articular index, and impairment were all shown to positively correlate with CRP levels, as were the prevalence of the disorder, synovial histology improvements, and radiographic advancement [62]. To diagnose RA, monitor the course of the condition, and forecast the prognosis of joint injuries, CRP has been found to be a helpful marker [63]. ESR is a common laboratory test that gauges how quickly the patient’s blood erythrocytes are settling inside a test tube. Red blood cell coagulation results from elevated amounts of fibrinogen in the blood during inflammatory responses, tumors, and autoimmune diseases [64].

To identify the diagnosis and monitor the development of the condition, patients with RA have also been prescribed ultrasound and magnetic resonance imaging (MRI) [65]. The power of grayscale with ultrasound examination of inflammatory joints, allows Doppler imaging of synovial proliferation, active inflammation, and neo-angiogenesis [66]. Furthermore, ultrasound can detect bone erosions [67], as well as subclinical synovitis, which can lead to radiographic disease worsening even when the patient appears to be in clinical remission [68].

The advantages of ultrasonography include its availability, affordability, lack of side effects, and non-invasive real-time imaging capabilities [69]. Also, MRI techniques, on the other hand, are a highly sensitive diagnostic tool for detecting synovial hypertrophy and pannus formation prior to the onset of bone erosion [70].

### Medication of RA

Traditional synthetic disease-modifying antirheumatic drugs (DMARDs) include TNF antagonists, anti-B cell, anti-T cell, and anti-IL6 antibodies, as well as methotrexate (MTX), sulfasalazine, leflunomide, and hydroxychloroquine [71]. MTX is the most often given drug for RA despite having an immunosuppressive effect and antiinflammatory qualities, and is reasonably priced. However, MTX has several restrictions because of toxicity. MTX has been related to immunological toxicity, cardiovascular toxicity, gastrointestinal toxicity, developmental toxicity, urinary toxicity, integumentary toxicity, and neurotoxicity [72]. Thus, the search for better and less expensive RA therapeutic agents is needed, so we will focus on this point in our review.

## Adenosine deaminase (ADA) a key inflammatory enzyme

The primary enzyme responsible for the RA pathway’s inflammation, ADA (EC 3.5.4.4), is a small, monomeric 40 kDa enzyme with 363 amino acid residues [73]. Three different isoforms of ADA exist in humans: ADA-1, ADA-2, and ADA-3. ADA-1 also exists in a low molecular weight form in addition to a complex with the ADA-binding protein CD26. The enzymatic mechanism of ADA-2 and ADA-1 are the same. It has a complex multi-domain design and is a homodimer of 114 kDa [74]. ADA-2 has only been found in eukaryotic and multicellular organisms, and it has been dubbed an ADA growth factor (ADGF). ADA-2 is mostly localized in extracellular space. The protein ADA adopts a triose phosphate isomerase (TIM) barrel structure, folding up and including eight periphery helices, according to X-ray crystallographic research. Five additional helices cause the barrel’s regularity to be decreased.

The enzyme’s active site is located and firmly embedded on the C-terminal side of the barrel. In the deepest portion of what seems to be a funnel-shaped pocket, a catalytic zinc ion is tightly bound. The zinc ion is needed by the enzyme’s mechanism, which permits the addition elimination reaction of ADA catalyzes. The intermediate tetrahedral transition state is produced when the water molecule transfers to its hydroxyl group stereo specifically in the C6 position of adenosine. The inosine product is produced following the intermediate’s subsequent ammonia has been lost. Additionally, the zinc ion has a significant structural role, since its absence causes structural changes that spread throughout the ADA and significantly reduce its stability (Fig. 8).

The regulating role of ADA in immune system activity was of interest as evidence that the major cause of reduced T- and B-cell function is a congenital deficit of this enzyme. It affects 20–30% of people with severe combined immunodeficiency disease (SCID). These results highlighted the critical role of ADA in the development and function of the immune system, as seen in Fig. 7, along with significant research activities targeted at clarifying the role of purine metabolism in immune cell activity. As a result, it has been discovered that ADA regulates a variety of immune cell types, including neutrophils, macrophages, lymphocytes, and dendritic cells [75].

### Role of ADA in inflammatory disorders

The function of ADA in the pathogenesis of RA illness has gained attention because of its unique immunological characteristics. The circulating mononuclear cells from patients with RA had far lower levels of ADA than cells from healthy individuals. On the other hand, the synovial effusions of those with RA contained high quantities of this enzyme activity [56].

As well as this, synovial fluid from those with reactive arthritis, juvenile chronic arthritis, chronic seronegative polyarthritis, and seropositive RA showed signs of ADA activation. The presence of ADA in synovial fluid exhibited a strong correlation with the disease’s systemic activity, as measured by hemoglobin concentration and erythrocyte sedimentation rate. This finding suggests that ADA activity should be evaluated as an additional criterion for judging the severity of joint inflammation. The enzyme activity was highest in the lymphocytes and monocytes of individuals with RA, and ADA-2 was the isoform that was only expressed in monocytes. Additionally, any possible connections between enzymatic activity in synovial fluid and the amount of matrix metalloproteinase-9 (MMP-9) present were elucidated, as well as the consequences of patients with RA having elevated ADA isozyme activity [76].

These results were confirmed by the fact that patients with RA had increased ADA activity in their synovial fluid and by the fact that their data showed strong positive correlations between MMP-9 and ADA isoforms [77]. The effects of MTX on a variety of enzymes, including ADA, hypoxanthine–guanine phosphoribosyltransferase, purine nucleoside phosphorylase, and 5′-nucleotidase. After taking the drugs, they saw a considerable reduction in all purine’s enzymatic activity. The appraisal of these measures as useful biochemical markers in patients with RA is further supported by prior research, which demonstrated a robust and proportional association between total blood ADA and ADA-2 activity and the degree of inflammation [78].

## Inhibitors of ADA activity

**Fig. 9:**
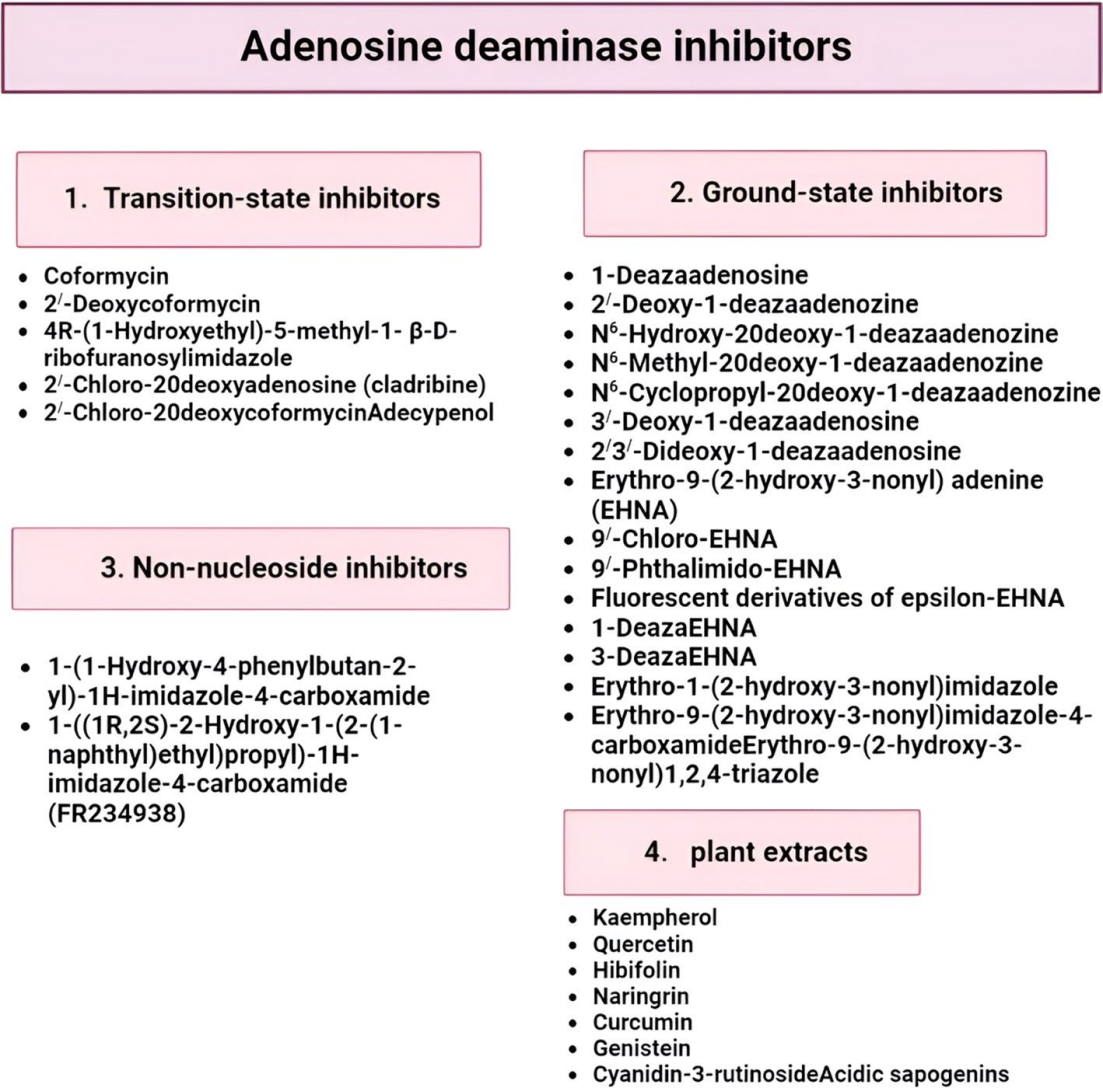
The main classes of adenosine deaminase inhibitors

ADA inhibitors come in four main varieties: transition state, ground state, non-nucleoside, and plant extracts. The tetrahedral intermediate produced by the ADA-catalyzed deamination process shares structural similarities with transition-state inhibitors [79]. The third class of derivatives, known as non-nucleoside inhibitors, was specifically composed of a set of imidazole-4-carboxamides produced and synthesized by Terasaka and colleagues at Fujisawa Pharmaceutical Company, which are equivalent to ground-state compounds [80]. Additional chemicals that successfully inhibit ADA activity include a wide range of medicines and phenolic compounds found in plants, such as flavonoids. The kinds of ADA inhibitors and the compounds that prevent ADA from interacting with cell surface proteins will be discussed in the review’s subsequent sections, as seen in Fig. 9.

### Transition-state inhibitors

#### Coformycin and deoxycoformycin analogs

In the sections that follow, we will go through the various ADA inhibitor classes and compounds that prevent ADA from interacting with cell surface proteins (Fig. 9) [81]. The two substances that effectively limit ADA activity the most frequently are the transition-state inhibitors. Their extraordinarily lengthy, practically irreversible, and tightly binding interactions with the enzyme are thought to be the reason for their efficiency [82]. The tetrahedral carbon (C8) in both variants has a hydroxyl group attached to it. The stereochemistry here has a big impact on potency; the 8*R*-diastereomer is almost 107 times stronger than the 8*S* equivalent [83].

### Ground-state compounds

#### Deaza- and dideazaadenosine derivatives

Compared with 7-deaza (tubercidin) and 1,7-dideazaadenosine, which are absolutely inert, 3-deaza and 1,3-dideazaadenosine are only weak inhibitors [84]. Although 1-deazaadenosine retains all molecular recognition properties when used as a substrate for ADA, it is not deaminated because it lacks the catalytically required N1-protonation [81]. A chlorine atom at position 2 decreased the inhibitory action of ADA. The compounds became more ADA resistant when a chlorine atom was added to the substrates in this location [85]. The 20-deoxyribose derivatives produced good inhibitory effects when hydroxyl, methyl, and cyclopropyl groups were substituted at the N6 position [86].

#### Erythro-9-(2-hydroxy-3-nonyl) adenine compounds

When adenine is connected at the N9 position to a chiral hydroxy nonyl chain, erythro-9-(2-hydroxy-3-nonyl) adenine (EHNA) is produced. The ADA inhibitor is a semi-tight one that first exhibits conventional competitive inhibition before successively rearranging the enzyme and tightly form ADA-inhibitor complex [81].

### Non-nucleoside inhibitors

Despite the above-mentioned drugs’ success in preventing ADA activity, their poor pharmacokinetics continue to prevent their broad use. However, fresh non-nucleoside ADA inhibitors have recently been created, such as a family of imidazole-4-carboxamides [81]. The molecular interactions between 1-deazaadenosine and murine ADA were the focus of these newly discovered compounds [87]. Additionally, a 1-(1-hydroxy-4-phenylbutan-2-yl)-1H-imidazole-4-carboxamide with good pharmacokinetics (oral bioavailability) was created [84].

### Plant extracts

In the typical person’s diet, flavonoids and phenolic compounds are found in plants, fruits, and vegetables. Numerous pharmacological effects of these plant phenolic and flavonoid compounds to modestly decrease of ADA activity had been studied [88]. The increase in endogenous adenosine that emerges from these drugs’ ability to inhibit competitive ADA may have some positive effects. Studies on the structure–activity correlation of these medicines and ADA indicate that the inhibitory effect requires the presence of the hydroxyl group at the three positions of the chromone molecule. There is a suggestion that the hydroxyl groups on the side of the phenyl ring are also significant [80].

## Natural product

Natural remedies have traditionally been used to treat infectious diseases and are now accepted cures for a wide range of illnesses [89]. Over the past 10 years, natural treatment has become widely accepted and the public has become more interested in it. As a result, herbal medications are now sold not only in drugstores but also in supermarkets and grocery shops. In Africa and other underdeveloped nations, almost 80% of people still use traditional herbal treatments to cure illnesses because they are more readily available and less expensive than manufactured drugs [90]. They also have antiinflammatory, spasmolytic, antioxidants sedative, antimicrobial, disinfectants, anti-diabetic, and immunostimulant properties against a variety of health problems [91].

### Quercetin

Quercetin is the name of the major polyphenolic flavonoid that may be found in berries, lovage, capers, cilantro, dill, apples, and onions. It belongs to one of the six subclasses of flavonoids [92]. It is completely soluble in lipids and alcohol and is colored yellow. It is, however, hardly soluble in hot water and insoluble in cold water. The word “quercetin” is derived from the Latin word “quercetum,” which means “oak forest.” Additionally, it belongs to the flavanol class, which the human body does not make [93]. The designations C_15_H_10_O_7_ and 2,3,5,7-trihydroxy-2,(3,4-dihydroxyphenyl)chromen-4-one, respectively, were assigned to the quercetin by the International Union of Pure and Applied Chemistry (IUPAC). One of the most important plant chemicals, quercetin is used medicinally to treat a variety of conditions, including arthritis, rheumatoid arthritis, allergic arthritis, metabolic illnesses, and inflammatory diseases [94].

#### Antiinflammatory effects of quercetin

Recently, quercetin proved its antiinflammatory activity through direct inhibition to ADA in RA rat model [95]. Moreover, several in vitro studies elucidated that quercetin could inhibit the generation of TNF-α, which is mediated by lipopolysaccharide (LPS) in macrophages and IL-8-induced LPS in lung A549 cells [96]. The capacity of quercetin to inhibit TNF-α and IL-1 levels of LPS-generated mRNA results in a reduced degree of apoptotic neuronal cell death produced by microglial activation. Quercetin prevents the synthesis of inflammatory enzymes such as cyclooxygenase (COX) and lipoxygenase (LOX) [97].

**Fig. 10:**
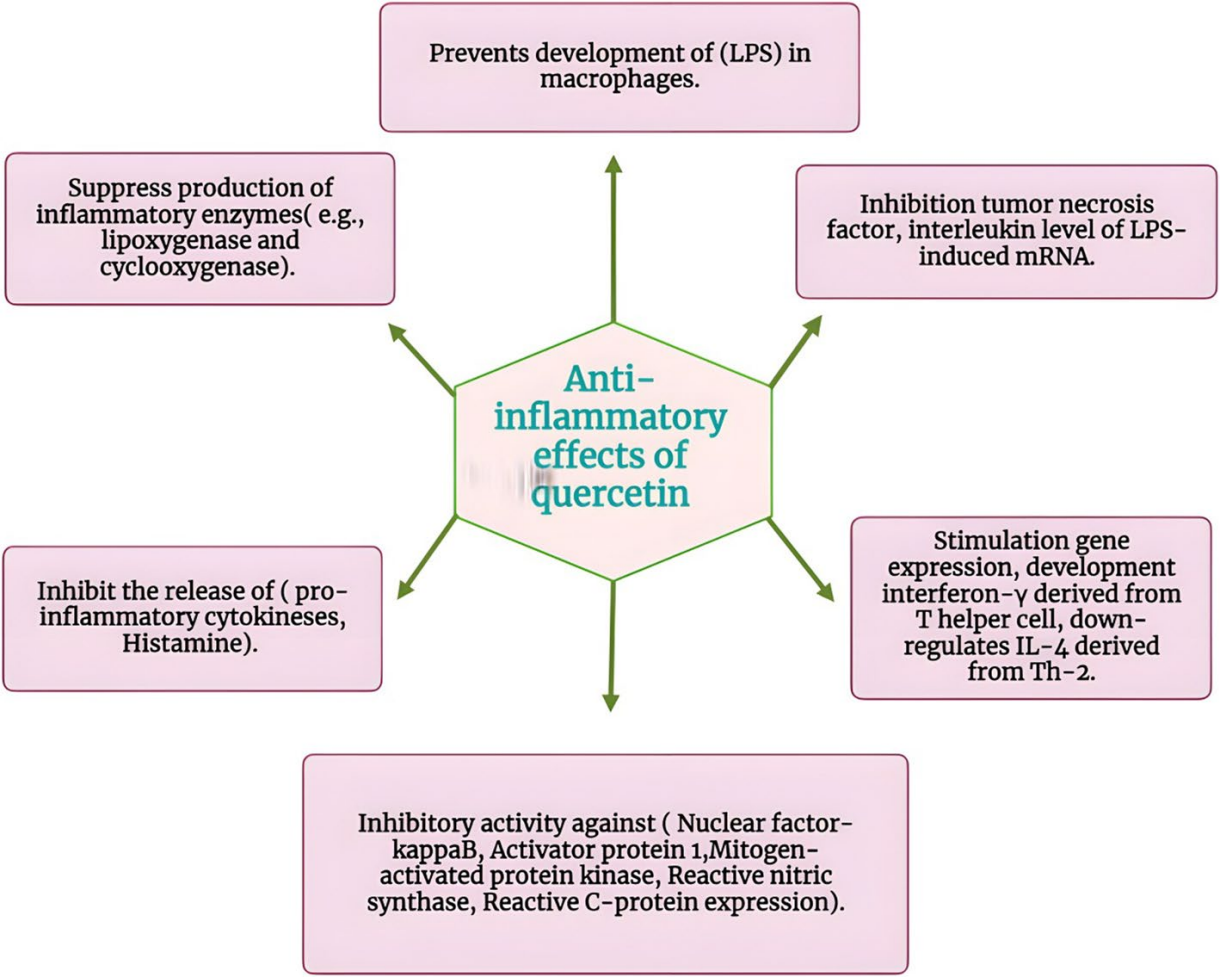
Antiinflammatory effects of quercetin

Additionally, it may limit the generation of tryptase, histamine, and proinflammatory cytokines by mast cells created from human umbilical cord blood; this protection is most likely brought about by the reduction of calcium influx and the suppression of phosphoprotein kinase C (PKC) [98] (Fig. 10).

Quercetin has demonstrated potent antiinflammatory activity with higher absorption through the skin’s surface in rats [99]. According to numerous studies, quercetin blocks the expression of vascular cell adhesion molecules (VCAM-1), intracellular cell adhesion molecules (ICAM-1), and E-selectin in human umbilical vein endothelial cells, as well as the secretion of iNOS, IL-1, and TNF induced by bacterial lipopolysaccharide (LPS) in macrophages, and RAW2647 cells. In non-alcoholic steatohepatitis (NASH) mice, quercetin and its glycoside rutin were shown to reduce TNF-α and IL-6 inflammatory markers [100] (Fig. 10).

Olabiyi et al., revealed that, with an IC_50_ value of about 0.00400005 mg/ml, quercetin had the strongest effect in inhibiting ADA [101]. The histological study supports all quercetin dosages’ efficacy in lowering edema development and the inflammatory response. This is in line with other studies that discovered quercetin reduced inflammatory effects on neutrophil activation and synovial cell activity [102].

Quercetin exerts antiinflammatory properties by regulating inflammatory cytokine production mediated by macrophages and T lymphocytes, as demonstrated by the finding that doses of quercetin (20 M and 40 M) could lower IFN levels in supernatants from activated Th cells cultured with either rutin or quercetin [103].

#### Inhibitory mechanism of adenosine deaminase (ADA) by quercetin (QUE) for RA treatment

RA is an autoimmune disease. It is well known that T and B lymphocytes are essential to the etiology and progression of RA [104]. Furthermore, it has been demonstrated that joint deterioration and an aggravation of clinical symptoms are associated with autoantibodies in patients with RA [105, 106].

**Fig. 11:**
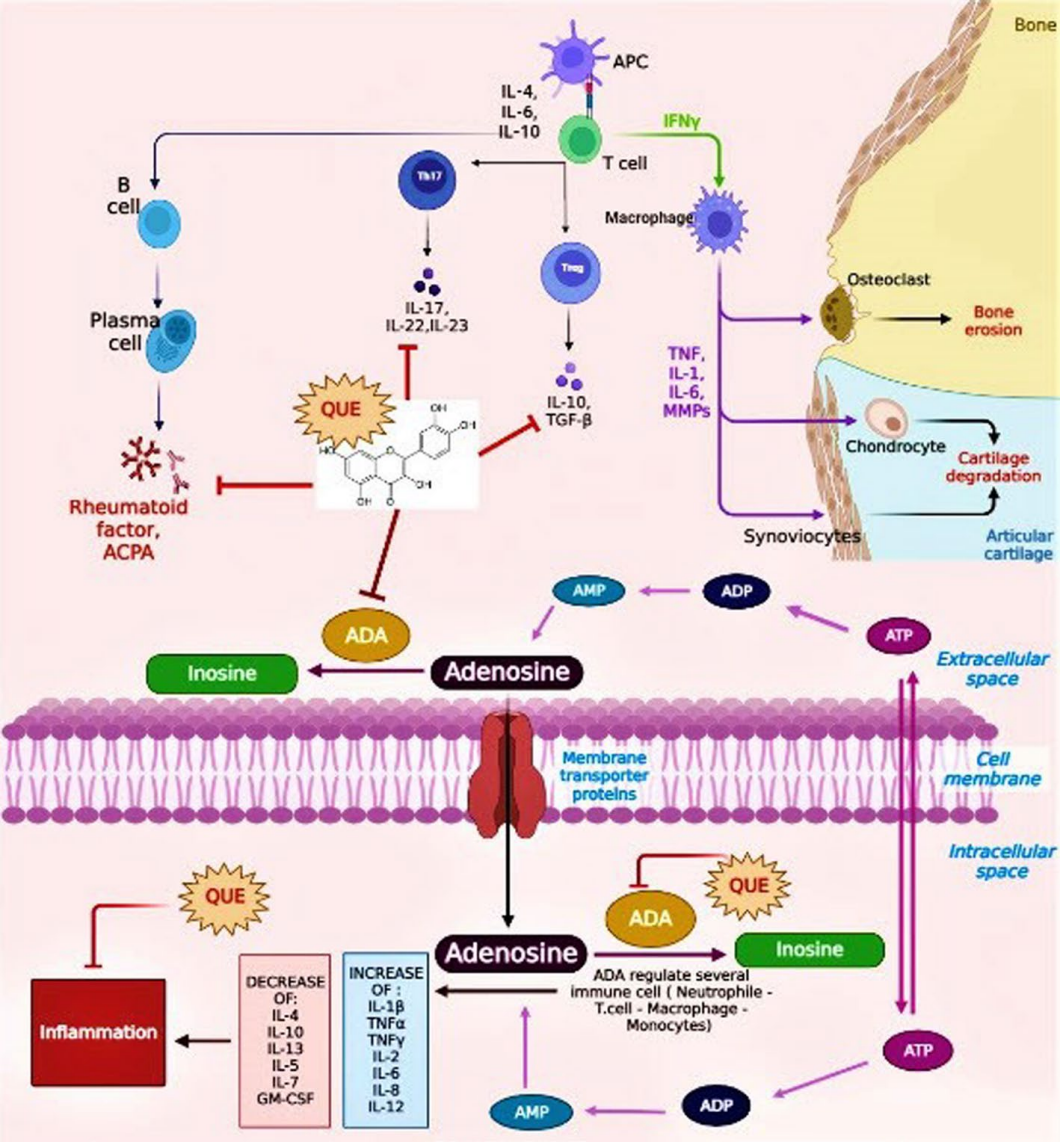
Mechanistic role of quercetin as inhibitor for adenosine deaminase enzyme for the treatment of rheumatoid arthritis

Adenosine deaminase (ADA) is an essential enzyme in purine metabolism, it converts adenosine to inosine to control intra- and extracellular adenosine concentrations [107, 108]. Adenosine is a significant purine that interacts with receptors and controls a wide range of physiological processes [109, 110]. Adenosine and subsequently adenosine deaminase could have either pro- or antiinflammatory effects on joints tissues [111]. Extracellular adenosine concentrations are typically less than 1 μM (30–200 nM) under normal settings, but they can rise to 100 μM in hypoxic and inflammatory situations [112]. Under low energy charge conditions, intracellular ATP breakdown is the primary source of extracellular adenosine [113, 114], which is subsequently stored and exported out of cells via equilibrate nucleoside transporters instead of being deaminated to inosine right away [115]. Moreover, adenosine nucleotides released into extracellular space can hydrolyze to form additional cellular adenosine under cellular stress conditions as shown in Fig. 11 [116]. Furthermore, it has been documented that adenosine deaminase activity increases in several disorders inside the body [117]. Therefore, the inhibition of this inflammatory key enzyme can significantly affect the clinical progression treatment of numerous diseases especially RA [118, 119].

The pathophysiology of RA involves lymphocytes that contain ADA with abnormal activity [120, 121]. According to the previous literature point of view, QUE has an inhibitory effect on the activity of lymphocytic ADA activity [122–124] as shown in Fig. 11.

This inhibitory effect is dieted by reduced the adenosine elevated levels through restoration of T-cell homeostasis [125–128], regulation of Th17 cell differentiation [129], regulation of Th17/Treg-related cytokine levels, reduction of autoantibody production, and regulation of nucleoside triphosphate diphosphohydrolase (NTPDase) activities [128, 130]. Collectively, QUE established the immune-regulatory effect and is considered one of the most important natural candidates that can used for RA therapy [131, 132] as mentioned in Fig. 11.

## Conclusion

Rheumatoid arthritis (RA) is an autoimmune disease that involves the immune system, particularly T and B lymphocytes, in its etiology and progression. Joint deterioration and worsening clinical symptoms in patients with RA are associated with autoantibodies. Adenosine deaminase (ADA), an essential enzyme in purine metabolism, plays a role in controlling adenosine concentrations and can have pro- antiinflammatory effects on joint tissues. Inhibition of ADA can potentially impact the clinical progression and treatment of RA. Intracellular ATP breakdown is primary source of extracellular adenosine, which increases in hypoxic and inflammatory condition. The pathophysiology of RA involves lymphocytes that contain ADA. Therefore, inhibition of lymphocytic ADA activity has been shown to have an immune-regulatory effect on such diseases. Quercetin (QUE) is considered an important natural candidate for RA therapy due to its immune-regulatory effect, as it inhibits lymphocyte ADA activity and reduce elevated adenosine. Also, QUE has the potential effect in restoring T cell homeostasis, regulating Th17 cell differentiation, and reducing autoantibody production. Ultimately QUE can be used as a potent candidate in the treatment of RA disease.

## Data Availability

The datasets used and/or analyzed during the current study are available from the corresponding author on reasonable request.

## Abbreviations

ADA: Adenosine deaminase
CD4^+^: Cluster of differentiation 4
COX: Cyclooxygenase
CRP: C-reactive protein
CXCR4: Alpha-chemokine receptor specific for stromal-derived factor 1
DC: Dendritic cell
DMARDs: Synthetic disease-modifying antirheumatic drugs
ESR: Erythrocyte sedimentation rate
GBD: Global Burden of Disease
GM-CSF: Granulocyte-macrophage colony-stimulating factor
ICAM-1: Intracellular cell adhesion molecules
IFN: Interferons
IL-1: Interleukin 1
IL-6: Interleukin 6
LOX: Lipoxygenase
LPS: Lipopolysaccharide
MCP-1: Monocyte chemoattractant protein 1
M-CSF: Macrophage colony-stimulating factor
MMP-1: Matrix metalloproteinase 1
MMP-9: Matrix metalloproteinase 9
MRI: Magnetic resonance imaging
MTX: Methotrexate
PKC: Phosphoprotein kinase C
RA: Rheumatoid arthritis
RANK-L: Receptor activator of nuclear factor kappa-β ligand
SCID: Severe combined immunodeficiency disease
SNPs: Single nucleotide polymorphisms
TIM: Triose phosphate isomerase
TLRs: Toll-like receptors
TNF-α: Tumor necrosis factor alpha
VCAM-1: Vascular cell adhesion molecules
VEGF: Vascular endothelial growth factor
